# Advances in Chairside and Restorative Dental Materials: A Systematic Review of Material Properties, Adhesive Strategies, and Long-Term Performance

**DOI:** 10.3390/jfb17080381

**Published:** 2026-08-03

**Authors:** Razvan Flueras, Ioana Elena Lile, Diana Marian, Ramona Amina Popovici, Călin Muntean, Radu Dumitru Moleriu, Lavinia Cristina Moleriu, Norina Consuela Forna, Ramona-Camelia Anculia, Ovidiu Motoc, Liana Todor

**Affiliations:** 1 Doctoral School, “Victor Babeș” University of Medicine and Pharmacy Timișoara, 300041 Timișoara, Romania; razvan.flueras@umft.ro; 2 Faculty of Dental Medicine, Vasile Goldiș Western University of Arad, 86 Liviu Rebreanu Street, 310414 Arad, Romania; lile.ioana@uvvg.ro; 3 Department I, Management and Communication in Dental Medicine, Faculty of Dental Medicine, Victor Babeș University of Medicine and Pharmacy of Timișoara E. Murgu Square, Nr. 2, 300041 Timișoara, Romania; 4 Department III—Functional Sciences, Medical Informatics and Biostatistics, “Victor Babeș” University of Medicine and Pharmacy, 300041 Timișoara, Romania; cmuntean@umft.ro (C.M.); radu.moleriu@umft.ro (R.D.M.); moleriu.lavinia@umft.ro (L.C.M.); 5 Department of Implantology, Removable Prostheses and Dental Prostheses Technology, Faculty of Dental Medicine, “Grigore T. Popa” University of Medicine and Pharmacy, 700115 Iasi, Romania; norina.forna@umfiasi.ro; 6 Discipline of Occupational Medicine, Faculty of Medicine, Victor Babes University of Medicine and Pharmacy of Timisoara, 300041 Timisoara, Romania; ramona.anculia@umft.ro; 7 Department of Dental Medicine, Faculty of Medicine and Pharmacy, University of Oradea, 10 1st Decembrie Street, 410073 Oradea, Romania; ovidiu.motoc@didactic.uoradea.ro (O.M.); liana.todor@uoradea.ro (L.T.)

**Keywords:** chairside CAD/CAM, lithium disilicate, zirconia, polymer-infiltrated ceramic network, resin nanoceramic, adhesive cementation, surface treatment, thermocycling, flexural strength, long-term performance

## Abstract

Background: Chairside CAD/CAM technology has reshaped restorative dentistry by enabling single-visit indirect restorations fabricated from glass-ceramics, zirconia, polymer-infiltrated ceramic networks (PICN), and resin nanoceramics. Despite a rapidly growing body of evidence, no recent systematic synthesis has integrated mechanical, adhesive, ageing-related, and clinical outcomes within a unified framework. Methods: This systematic review was conducted and reported in accordance with PRISMA 2020. A structured electronic search was performed in the Web of Science Core Collection and MEDLINE via PubMed for studies published between January 2021 and May 2026. The search was complemented by Google Scholar and reference-list screening. Eligible primary studies were selected according to predefined PICOS criteria and synthesised qualitatively because methodological and outcome heterogeneity precluded meta-analysis. Of the 444 records initially identified, 332 were screened after duplicate removal, and 31 primary studies met all eligibility criteria and were included in the qualitative synthesis. Review-level publications were used only for reference-list screening and contextual comparison and did not contribute data to the synthesis. Results: Laboratory studies indicated favourable flexural-strength profiles for lithium disilicate, advanced lithium disilicate, and Y-TZP zirconia, while PICN blocks showed comparatively favourable resistance to thermomechanical ageing. The limited available clinical evidence reported high short-term survival for chairside CAD/CAM restorations, but did not permit definitive comparisons between material classes. Across the predominantly in vitro bonding studies, hydrofluoric-acid etching followed by silanisation produced the most consistent bond-strength results for glass-ceramics, whereas Al_2_O_3_ air-abrasion combined with an MDP-containing primer showed favourable bond strength to zirconia. Universal adhesives showed promising laboratory performance on hybrid blocks but did not consistently match the performance of dedicated surface-conditioning protocols for glass-ceramics and zirconia. Conclusions: Material selection in chairside dentistry should consider intrinsic material properties, surface-conditioning requirements, and the planned cementation strategy. Standardised laboratory protocols and adequately powered prospective clinical studies with longer follow-up are required before definitive material-specific clinical recommendations can be established, particularly for advanced lithium disilicate and multilayer zirconias.

## 1. Introduction

The integration of intraoral scanning, computer-aided design, and computer-aided manufacturing (CAD/CAM), together with the development of glass-ceramic, zirconia, and polymer-based blocks, has enabled the fabrication and delivery of indirect restorations within a single clinical appointment [1,2,3,4]. Early work established the methodological framework and reference datasets for evaluating these materials [3,5]. Chairside CAD/CAM inlays, onlays, overlays, and crowns now compete with conventional layered composite restorations in cost, time, and predictability, while offering superior wear resistance, colour stability, and marginal integrity [6,7,8].

From a materials-science perspective, the chairside CAD/CAM portfolio is currently dominated by five major families (hereafter referred to collectively as “chairside CAD/CAM restorative materials”, or “blocks”), as follows: feldspathic/leucite-reinforced ceramics, lithium-(di)silicate-based glass-ceramics (including the recent advanced lithium disilicate, ALD, and zirconia-reinforced lithium silicate, ZLS), yttria-stabilised tetragonal zirconia polycrystals (3Y-, 4Y- and 5Y-TZP), polymer-infiltrated ceramic networks (PICN) and resin-based nanoceramic blocks [9,10]. Each class has been engineered to balance flexural strength, fracture toughness, translucency, water sorption and elastic modulus to match a specific clinical indication, ranging from anterior veneers to posterior monolithic crowns [11,12,13,14].

Beyond their intrinsic mechanical and optical properties, the performance of chairside CAD/CAM restorations is influenced by the adhesive interface between the tooth substrate, the luting material, and the restorative block. Surface-conditioning procedures—including hydrofluoric-acid etching, alumina air-abrasion, tribochemical silica coating, silanisation, and the application of MDP-containing primers—can substantially affect bond strength and durability [15,16,17,18]. Universal adhesives have been proposed to simplify these procedures; however, their effectiveness remains material-dependent, particularly when used with glass-ceramics, zirconia, and polymer-containing substrates [12,15,18].

Translating in vitro performance into clinical longevity remains challenging. In the oral environment, restorative materials are exposed to cyclic mechanical loading, thermal fluctuations, moisture, dietary acids, biofilm accumulation, prophylactic procedures, and bleaching agents. These factors may affect surface roughness, microhardness, colour stability, wear, and adhesive durability over time [19,20,21]. Moreover, laboratory studies differ substantially in specimen preparation, testing configuration, ageing procedures, and outcome measurement, thereby limiting direct comparisons between materials and the extrapolation of experimental findings to clinical practice. These factors act differentially across material families, as follows: PICN blocks preserve their microhardness after thermocycling better than nano-filled resin composites, whereas glass-ceramics may alter after prophylaxis or bleaching [22,23,24,25,26]; nonetheless, partial-coverage restorations still show high two-year survival, even with deep-margin elevation [26].

The literature published between 2021 and 2026 is heterogeneous in the materials investigated, experimental designs, ageing protocols, adhesive strategies, and reported outcomes. Previous reviews have generally addressed individual domains, such as mechanical behaviour, adhesive performance, or clinical survival. The present review was therefore designed as an integrative qualitative synthesis of these interrelated domains in the specific context of chairside CAD/CAM indirect restorations. This approach was intended to identify areas of convergence, explain sources of disagreement between studies, and clarify the extent to which laboratory findings are currently supported by clinical evidence.

Accordingly, this review aimed to do the following: (i) compare the mechanical and selected physico-chemical properties of contemporary chairside CAD/CAM restorative materials; (ii) critically evaluate the surface-conditioning and adhesive strategies investigated for each material family; and (iii) synthesise the available evidence on thermomechanical ageing and short-term clinical performance. Particular attention was given to methodological heterogeneity, the distinction between laboratory and clinical evidence, and the limitations affecting the translation of experimental findings into clinical recommendations.

## 2. Materials and Methods

### 2.1. Protocol and Reporting

This systematic review with qualitative synthesis was conducted and reported in accordance with the Preferred Reporting Items for Systematic Reviews and Meta-Analyses (PRISMA) 2020 statement [27,28]. Before study selection and data extraction, the review team established the research question, PICOS framework, eligibility criteria, search strategy, screening procedure, and data-extraction items. The protocol was subsequently registered retrospectively in the Open Science Framework on 13 July 2026, after completion of the review, under the DOI https://doi.org/10.17605/OSF.IO/26FCD. Because the registration was retrospective, no claim of prospective protocol registration is made. A meta-analysis was not undertaken because of substantial heterogeneity in study designs, material categories, specimen configurations, ageing protocols, outcome measures, and follow-up durations.

### 2.2. Research Question and PICOS Framework

The review question was as follows: “How do contemporary chairside CAD/CAM restorative materials differ in mechanical properties, adhesive performance, and resistance to thermomechanical ageing, and to what extent can these findings inform clinical material selection?”.

The review was designed as an integrative qualitative synthesis rather than as an exhaustive evaluation of a single outcome domain. Its scope was defined by three prespecified objectives, as follows: comparison of mechanical and selected physico-chemical properties, evaluation of material-specific adhesive strategies, and synthesis of ageing-related and short-term clinical evidence. The PICOS framework was operationalised as follows:

Population/specimens (P): adult patients receiving chairside CAD/CAM indirect restorations and, for laboratory studies, extracted human teeth or standardised restorative-material specimens intended to simulate indirect restorative applications.
Intervention (I): chairside CAD/CAM restorations fabricated from feldspathic, leucite-reinforced, lithium-(di)silicate or zirconia-reinforced lithium silicate ceramics, Y-TZP zirconia, polymer-infiltrated ceramic networks (PICN) or resin nanoceramic blocks.
Comparator (C): an alternative CAD/CAM material, surface-conditioning or cementation protocol, ageing condition, conventional indirect restorative approach, or non-aged baseline condition.
Outcomes (O): mechanical properties, including flexural strength, fracture resistance, fracture toughness, fatigue behaviour, and microhardness; physico-chemical and surface outcomes, including water sorption, solubility, roughness, and colour stability; adhesive outcomes, including shear, microshear, tensile, or microtensile bond strength; and clinical outcomes, including survival, marginal integrity, complications, and restoration failure.
Study design (S): primary in vitro experimental studies and prospective or retrospective clinical studies. Systematic reviews, meta-analyses, scoping reviews, narrative reviews, and other secondary research publications were not eligible for inclusion as studies, but their reference lists could be screened to identify potentially eligible primary investigations.

### 2.3. Information Sources and Search Strategy

A systematic electronic search was conducted in the Web of Science Core Collection and MEDLINE via PubMed. The eligibility period covered studies published from January 2021 through 31 May 2026, and only English-language full-text publications were considered. Only primary in vitro and clinical studies were eligible for inclusion in the qualitative synthesis. The search strategy combined terms related to chairside CAD/CAM restorative materials, mechanical and physico-chemical properties, adhesive performance, surface treatment, ageing, and clinical outcomes. Database-specific search strings were adapted to the syntax of each platform, and the complete Web of Science search strategy is reported below. The full bibliographic details of the primary studies included following the selection process [10,12,13,15,16,17,18,19,20,21,22,23,24,25,26,29,30,31,32,33,34,35,36,37,38,39,40,41,42,43,44] are provided in Table 1. The Web of Science Core Collection search was performed on 3 May 2026 and retrieved 303 records. The PubMed/MEDLINE search was performed on 10 June 2026 and retrieved 127 records. The electronic search was complemented by a hand search of Google Scholar and of the reference lists of the most recent systematic reviews, which identified 14 additional records. Therefore, a total of 444 records were identified before de-duplication.

Scopus was not searched separately because Web of Science Core Collection and MEDLINE via PubMed were considered complementary sources for the dental materials and restorative dentistry literature. Nevertheless, the absence of a separate Scopus search may have reduced retrieval sensitivity and is acknowledged as a limitation. Supplementary Google Scholar screening and reference-list searching were used to reduce, but could not eliminate, the risk of missing eligible studies.

TS = ((CAD/CAM OR “chairside CAD/CAM” OR “milled restoration*” OR inlay OR onlay OR overlay) AND (“restorative material*” OR ceramic* OR zirconia OR “hybrid ceramic*” OR “resin composite*” OR “lithium disilicate” OR “polymer-infiltrated ceramic”) AND (property* OR “mechanical propert*” OR flexural OR translucenc* OR “water sorption” OR “water absorption” OR ageing OR degradation OR “colour stability” OR biocompatibility) AND (adhesion OR bonding OR “bond strength” OR “surface treatment*” OR “adhesive strategy*” OR “self-etch” OR “etch-and-rinse” OR silane OR sandblasting OR “hydrofluoric acid”) AND (“long-term” OR “clinical performance” OR survival OR durability OR fracture OR failure OR microleakage OR wear)).

Document types were limited to original articles, reviews, and meta-analyses; the language was restricted to English. The complete WoS query and result set are publicly accessible at https://www.webofscience.com/wos/woscc/summary/57e34075-aaac-4235-91fc-dfc781b3319d-01b1e7b718/relevance/7, accessed on 3 May 2026). A complementary hand search of Google Scholar and the reference lists of the most recent systematic reviews [6,8] was conducted to retrieve additional studies published in 2025–2026 that were not yet indexed.

The supplementary Google Scholar hand search used keywords and Boolean combinations adapted from the main database query. The first 200 results (sorted by relevance) were screened at the title and abstract level, and screening was stopped when no new potentially eligible records appeared across three consecutive result pages. Together with reference-list searching of the most recent systematic reviews, this supplementary process identified 14 additional records that were not indexed in the primary databases, of which 3 were ultimately included after full-text assessment and de-duplication. Although the Web of Science search was executed on 3 May 2026 and the PubMed/MEDLINE search on 10 June 2026, the a priori eligibility window was restricted to studies published between January 2021 and May 2026; the PubMed search was deliberately extended to 10 June 2026 only to capture records bearing an official May 2026 publication date that had been indexed with a short delay, and no study published after 31 May 2026 was eligible or included.

### 2.4. Eligibility Criteria

Inclusion criteria were as follows:
  - original primary in vitro experimental studies or prospective or retrospective clinical studies evaluating chairside CAD/CAM indirect restorative materials;
  - evaluation of at least one mechanical, physico-chemical, surface, adhesive, ageing-related, or clinical outcome relevant to the review question;
  - publication between January 2021 and 31 May 2026;
  - publication in English;
  - availability of the full text.
Exclusion criteria were as follows:
  - studies focused exclusively on direct restorative materials;
  - studies evaluating metal-ceramic or cast-metal indirect restorations;
  - systematic reviews, meta-analyses, scoping reviews, narrative reviews, and other secondary research publications;
  - conference abstracts, editorials, letters, opinion papers, case reports, and case series without comparative data;
  - in vivo animal studies;
  - studies involving primary dentition only;
  - studies in which the CAD/CAM material or restorative indication was not clearly identified;
  - duplicate reports or publications reporting overlapping datasets without additional relevant outcomes.

### 2.5. Study Selection and Data Extraction

After de-duplication, two reviewers independently screened titles and abstracts. Potentially eligible reports were retrieved in full text and assessed against the eligibility criteria. Disagreements were resolved through discussion and, when necessary, consultation with a third reviewer.

Data were extracted using a standardised form that recorded the study author and year, study design, sample or specimen characteristics, material family and commercial product, comparator, surface-conditioning and cementation protocol, ageing procedure, test method, relevant standards, primary outcomes, and principal numerical findings.

The complete bibliographic characteristics of the primary studies included in the qualitative synthesis, including authorship, publication year, article title, journal, document type, DOI, and Web of Science accession number, are presented in Table 1.

When multiple reports appeared to arise from the same study population or experimental dataset, the reports were compared by author group, sample characteristics, materials, methods, and recruitment or testing period. Overlapping results were included only once, while reports providing distinct outcomes or follow-up periods were cross-referenced and treated as companion publications.

#### Use of Review-Level Publications for Supplementary Searching and Contextualisation

Systematic reviews, meta-analyses, scoping reviews, and narrative reviews were not eligible for inclusion in the qualitative synthesis and did not contribute outcome data. Their reference lists were screened to identify potentially eligible primary studies that may not have been retrieved through the electronic searches; selected review-level publications were also cited in the Discussion to compare the present findings with previously synthesised evidence. They were not counted among the included studies, entered into the data-extraction tables, or subjected to the risk-of-bias assessment. Consequently, no primary study result was counted both directly and through a secondary publication.

### 2.6. Descriptive Numerical Summary of Extracted Data

Because substantial heterogeneity in study design, specimen geometry, test configuration, ageing procedures, outcome definitions, and reporting methods precluded meta-analysis, the extracted numerical data were summarised descriptively. No pooled effect estimates were calculated.

When comparable numerical results were available, the values were reported as study-specific results or as the minimum–maximum range observed across the relevant primary studies. Any central or mid-range values used for graphical presentation were unweighted descriptive values intended solely to facilitate visual comparison between material categories. They were not calculated using meta-analytic methods and were not weighted according to sample size, standard error, variance, study quality, or risk of bias.

Accordingly, all graphical and tabular numerical summaries should be interpreted as qualitative descriptive benchmarks rather than pooled estimates or comparative effect measures. The study-level summary of the methodological quality and overall risk-of-bias assessments is presented in Supplementary Table S1 [10,12,13,15,16,17,18,19,20,21,22,23,24,25,26,29,30,31,32,33,34,35,36,37,38,39,40,41,42,43,44].

### 2.7. Risk-of-Bias and Quality Appraisal

The methodological quality and risk of bias of the included primary studies were assessed using design-specific instruments. The 30 in vitro experimental studies were evaluated using an adapted eight-item Sarkis-Onofre checklist. The assessed domains were randomisation or allocation of specimens, sample-size or power calculation, standardisation of specimen preparation, operator or assessor blinding, use of an appropriate control or reference group, adherence to manufacturer instructions, appropriateness of the statistical analysis, and performance of failure-mode, fractographic, or other validation analyses. Each fulfilled item received one point. Total scores of 7–8 were classified as low risk of bias, 5–6 as moderate risk of bias, and 0–4 as high risk of bias. The prospective clinical cohort study was assessed using the Newcastle–Ottawa Scale. Scores of 7–9 stars were interpreted as low risk of bias, scores of 4–6 stars as moderate risk of bias, and scores of 0–3 stars as high risk of bias. A detailed description of the appraisal instruments, assessed domains, scoring procedures, and thresholds used to classify the overall risk of bias is provided in Supplementary Table S2. Two reviewers independently performed the assessments. Disagreements were resolved through discussion and, when necessary, consultation with a third reviewer. Inter-rater agreement was evaluated using Cohen’s kappa. The study-level summary of the methodological quality and overall risk-of-bias assessments is presented in Supplementary Table S1, while the item-level judgements, total scores, and corresponding risk-of-bias classifications are reported in Supplementary Table S3.

## 3. Results

### 3.1. Study Selection and Bibliometric Overview

**Figure 1:**
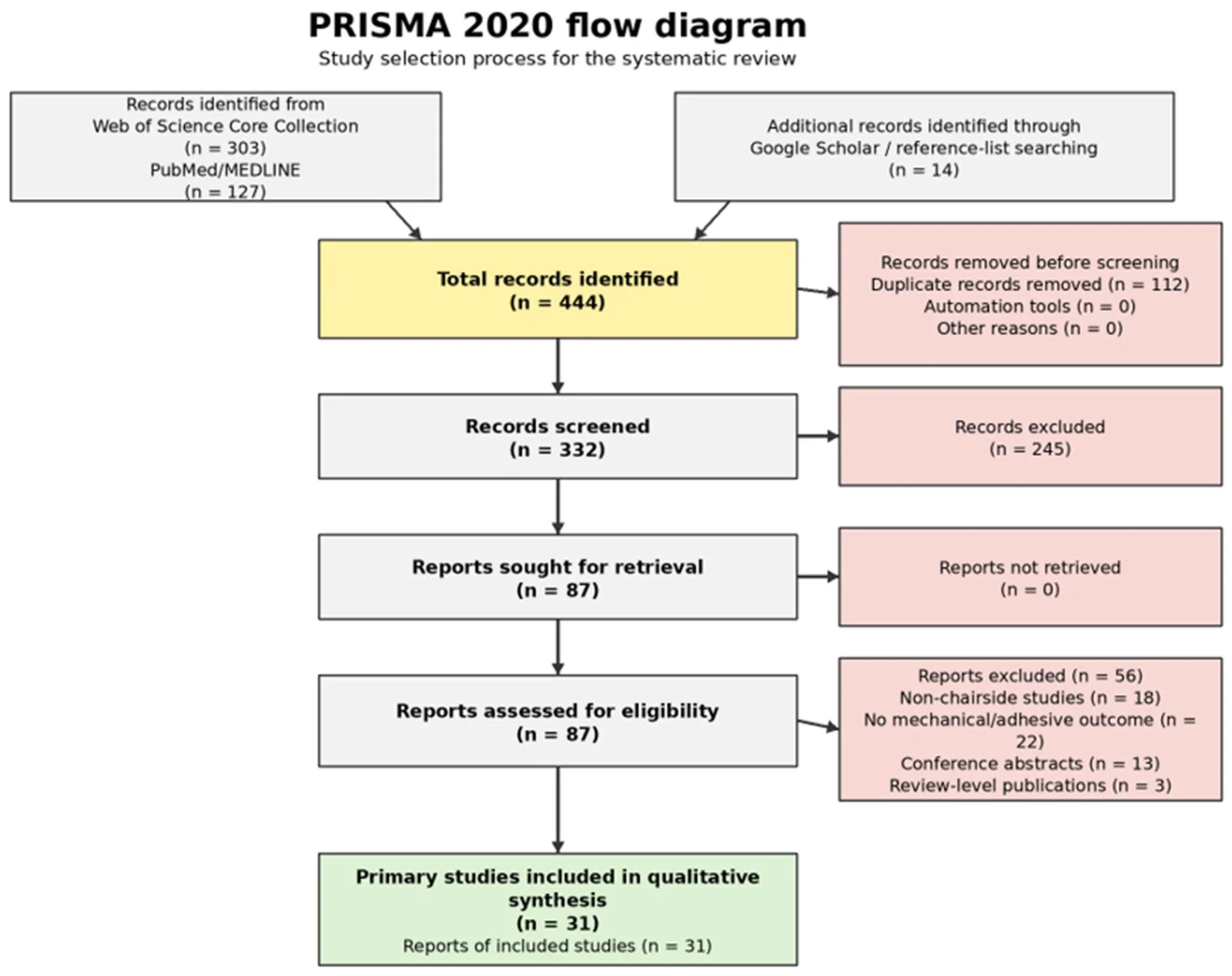
PRISMA 2020 flow diagram of the literature search and study-selection process. A total of 444 records were identified: 303 from the Web of Science Core Collection, 127 from MEDLINE via PubMed, and 14 through Google Scholar and reference-list screening. After removal of 112 duplicates, 332 records were screened, and 87 full-text reports were assessed for eligibility. Fifty-six reports were excluded, and 31 primary studies were included in the qualitative synthesis. Review-level publications were not eligible for inclusion but were used for supplementary reference-list screening and contextual comparison.

The database search identified 430 records, as follows: 303 from the Web of Science Core Collection and 127 from PubMed/MEDLINE. An additional 14 records were identified through Google Scholar and reference list searches. Thus, 444 records were identified before de-duplication. After removal of 112 duplicates, 332 records underwent title and abstract screening. Eighty-seven full-text reports were assessed for eligibility, of which 56 were excluded. Thirty-one primary studies met all eligibility criteria and were included in the qualitative synthesis. These comprised 30 in vitro experimental studies and one prospective clinical cohort study (Figure 1).

**Figure 2:**
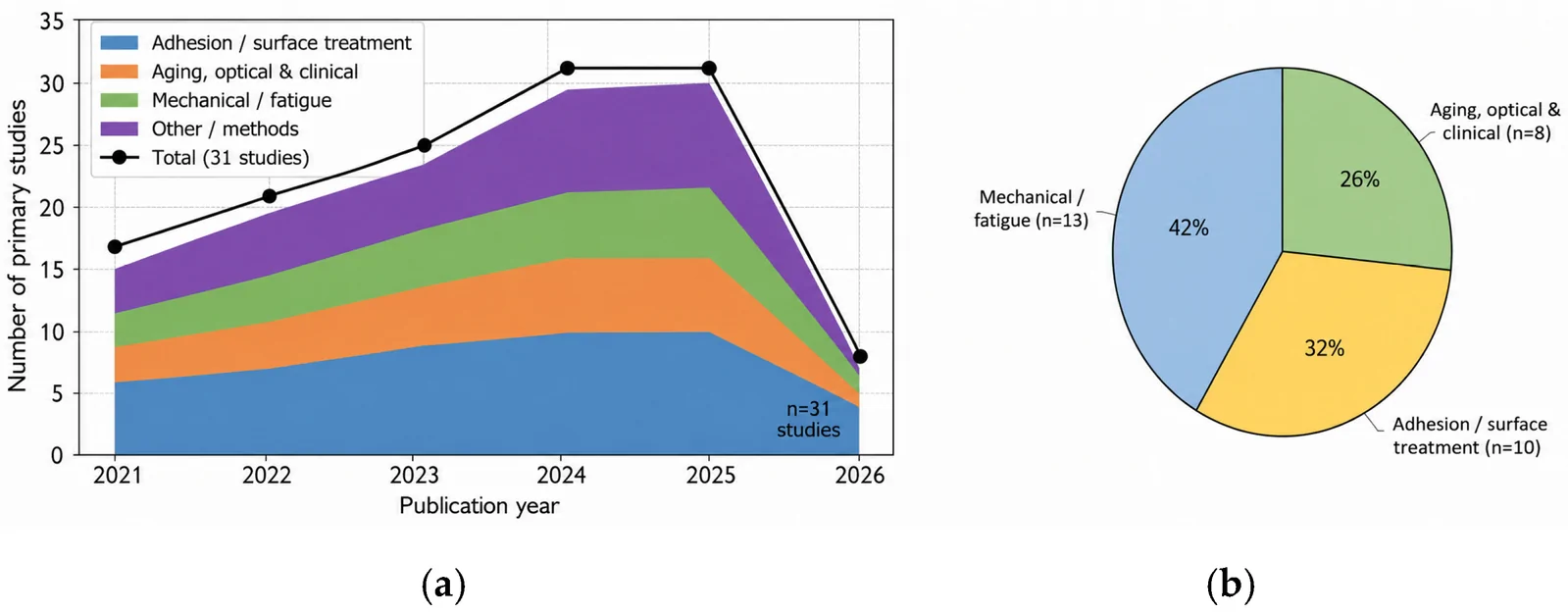
Distribution of the primary studies included in the qualitative synthesis: (**a**) publication year; and (**b**) principal outcome domain. The 2026 count is partial because the eligibility period ended on 31 May 2026.

The 31 included studies span all six years of the search interval and were published in journals such as Journal of Functional Biomaterials, Biomaterials Advances, Dental Materials, Materials, Journal of Dentistry, and Journal of Adhesive Dentistry. Figure 2 summarises the temporal trend and topical distribution of the included literature.

Studies were grouped according to their primary outcome domain, as follows: mechanical and fatigue behaviour (*n* = 13), adhesion and surface treatment (*n* = 10), and ageing, optical, and clinical performance (*n* = 8). The most-investigated material families were lithium disilicate (LS_2_) and ALD ceramics (n = 12), Y-TZP zirconia (*n* = 9), PICN (*n* = 8), and resin nanoceramic blocks (*n* = 7); some studies examined more than one family in parallel.

In accordance with the strategy defined in Section Use of Review-Level Publications for Supplementary Searching and Contextualisation, all descriptive numerical data reported below derive exclusively from the primary studies; Selected review-level publications were cited only for comparative positioning and contextualisation in the Discussion and did not contribute data to the qualitative synthesis.

### 3.2. Risk-of-Bias and Quality Appraisal Results

The risk-of-bias assessment is summarised in Supplementary Table S1. Among the 30 included in vitro studies, six were classified as having a low risk of bias, 20 as having a moderate risk of bias, and four as having a high risk of bias. The single prospective clinical cohort study was classified as having a low risk of bias according to the Newcastle–Ottawa Scale. The most consistently fulfilled criteria were standardised specimen preparation, adherence to manufacturer instructions, and use of appropriate statistical analyses. The most frequently unmet criteria were an a priori sample size or power calculation and operator or assessor blinding. Inter-rater agreement was substantial (Cohen’s κ = 0.81).

### 3.3. Classification, Composition, and Indications

**Figure 3:**
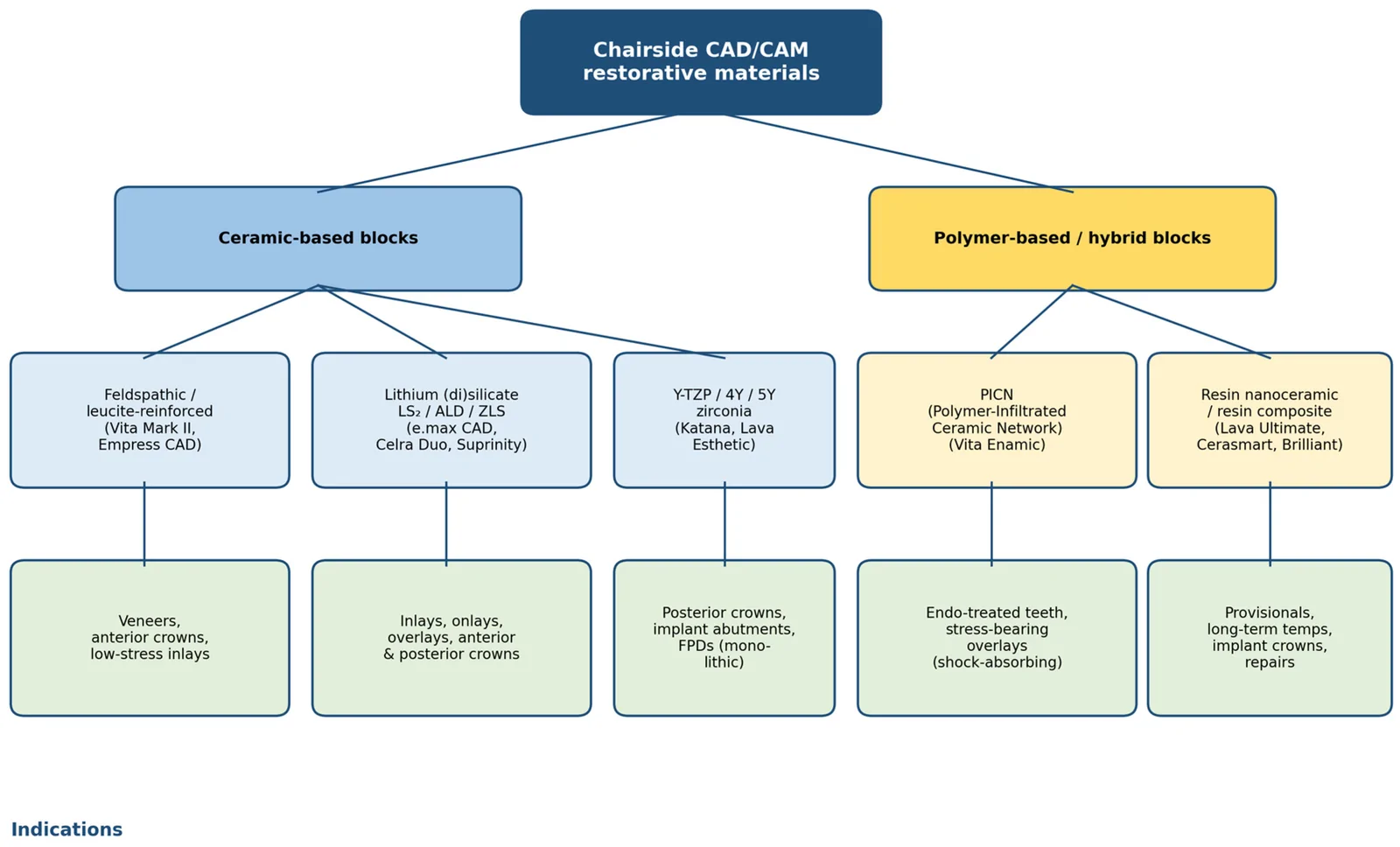
Classification scheme of contemporary chairside CAD/CAM restorative materials, with representative commercial brands and main clinical indications.

Contemporary chairside CAD/CAM materials can be grouped into two main families—ceramic-based and polymer-based/hybrid—each subdivided according to microstructure and clinical indication (Figure 3). Ceramic blocks include feldspathic/leucite-reinforced ceramics, lithium-(di)silicate-based glass-ceramics (LS_2_, ALD, ZLS), and polycrystalline yttria-stabilized zirconia (3Y-, 4Y-, 5Y-TZP). Polymer-based/hybrid blocks include polymer-infiltrated ceramic networks (PICN, e.g., Vita Enamic) and highly cross-linked resin nanoceramic blocks (e.g., Lava Ultimate, Cerasmart). Table 2 summarises the composition, microstructure, and main clinical indications of the most representative blocks marketed for chairside fabrication, while Figure 3 presents the corresponding classification scheme.

### 3.4. Mechanical and Physico-Chemical Properties

The mechanical performance of CAD/CAM blocks was assessed using the mechanical and physico-chemical outcomes reported in the eligible primary studies. Descriptive numerical findings extracted exclusively from these studies are presented in Table 3. Because the included studies differed substantially in material brands, specimen geometry, testing methods, and ageing conditions, the values were not statistically pooled. Recent investigations have refined these benchmarks, as follows: Fouda et al. [25] demonstrated that thermocycling reduces the fracture toughness of fully crystallised aluminosilicate blocks by ~12% and that surface glazing only partially mitigates this effect, while Kawajiri et al. [36] confirmed that PICN nanocomposites match human enamel in both Vickers hardness (~3.0 GPa) and flexural modulus (~30 GPa). Sismanoglu et al. [45] further reported that the relative performance of polymer-based CAD/CAM blocks is heavily dependent on the bonding-promoter chemistry rather than on bulk composition alone, while Shono et al. [35] documented a 25–30% increase in biaxial flexural strength once specimens were adhesively bonded to a resin substrate.

Yttria-stabilised zirconias remain in a class of their own, with flexural strengths roughly twice those of any glass-ceramic block; the recently introduced 4Y- and 5Y-TZP grades trade some mechanical performance for improved translucency, while preserving K_IC values above 3.5 MPa·m^0.5^ [8,46,47]. Among glass-ceramics, ALD blocks have closed the historical gap between lithium disilicate and zirconia, with reported flexural-strength values ranging from 445 to 520 MPa and microhardness comparable to e.max CAD [13,47]. Resin-matrix blocks exhibit the lowest moduli (12–35 GPa), which is often cited as advantageous for cuspal flexion in endodontically treated teeth [45,48], although their substantially higher water sorption (32–42 μg/mm^3^) raises concerns about long-term hygroscopic expansion and colour drift [22].

Glass-ceramics, and in particular feldspathic and lithium disilicate blocks, exhibit a more favourable balance between translucency and colour stability than polymer-based blocks [19,22,24,25] (Supplementary Table S4); the latter show consistently higher ΔE after thermocycling and external bleaching, in agreement with the recent simultaneous thermocycling–staining experiments by Elsahn et al. [22].

### 3.5. Thermomechanical Ageing and Surface Stability

Ageing is a critical step toward predicting clinical longevity. The included studies used a wide range of protocols, most often combining 5000–10,000 thermal cycles between 5 °C and 55 °C with 1.2–2.4 × 10^6^ mechanical cycles at 50–200 N (Table 4) [31,41].

Across the included thermocycling experiments [20,21,22,24], polymer-rich blocks consistently exhibited the largest decreases in Vickers microhardness and the largest increases in surface roughness, whereas glass-ceramics and zirconia were comparatively stable. Importantly, Fajardo et al. [20] reported that PICN retained 88% of its baseline microhardness after 10,000 TC, compared with only 70–75% for nano-filled resin composites—a finding that supports the growing clinical preference for PICN when a hybrid block is indicated. da Rosa et al. [21] further showed that prophylactic polishing with pumice or air-powder abrasion induces clinically negligible changes on monolithic zirconia and lithium disilicate, while measurably increasing surface roughness on leucite-reinforced and resin-matrix blocks. Surface conditioning post-crystallisation (polishing vs glazing) on ZLS ceramics, as investigated by Rea et al. [19], yielded significantly lower Ra values for the polished groups, supporting polishing as the preferred chairside finishing protocol. Complementary evidence from Patil and Jebaseelan [49] on milled zirconia and from Vulovic et al. [46] on implant-supported CAD/CAM substrates confirms that post-fabrication surface treatments influence not only roughness but also wettability and contact-angle behaviour, with direct implications for biofilm formation. Sahin et al. [24] further showed that simulated toothbrushing combined with surface treatments measurably affects both the colour stability and the surface roughness of CAD/CAM interim crown materials, underscoring the cumulative nature of intra-oral ageing. Machry et al. [34] reported that the foundation substrate beneath the restoration also modulates fatigue performance, particularly for polymer-infiltrated and resin-based blocks.

### 3.6. Adhesive Strategies and Surface Treatments

Bond strength to CAD/CAM blocks is governed by a synergistic interaction among the substrate’s chemical nature, the applied surface treatment, and the chosen primer/cement. Table 5 summarises the surface conditioning protocols recommended for each material family based on the evidence included [38,42,43,50,51,52,53,54]. Beyond the standard protocols, Kwak and Choe [14] demonstrated that combining HF etching with low-pressure Al_2_O_3_ air-abrasion produces a more homogeneous topography on lithium disilicate, while Pulido et al. [43] showed that prolonged application of self-etching primer (Monobond Etch and Prime) recovers up to 80% of the bond strength achieved with traditional HF + silane on LS_2_ and ZLS. Janson et al. [55] reported that self-adhesive resin cements remain inferior to MDP-doped dual-cure cements on silicate ceramics by 6–10 MPa. Yazigi et al. [53] highlighted the importance of saliva decontamination, demonstrating that bond strength to silicate and zirconia blocks drops by 25–35% after intra-oral try-in unless an appropriate cleaning agent is applied.

Across the included adhesion studies [12,15,16,18,38,39,41,42,43] (Supplementary Table S5), hydrofluoric acid etching followed by silanisation reproducibly yielded the highest and most stable bond-strength values on glass-ceramic blocks (26–34 MPa baseline; 20–30 MPa after 5000 thermocycles) [7,42]. For Y-TZP zirconia, alumina air abrasion (50 µm, 2–3 bar) combined with an MDP-containing primer increased SBS by 8–12 MPa compared with a universal adhesive alone [16,41,52]. The recent investigation of Yavuz et al. [16] on additively versus subtractively manufactured blocks confirmed that fine-particle Al_2_O_3_ followed by silane represents the most consistent protocol across both manufacturing routes. Universal adhesives (Vidal et al. [12]; Bashary et al. [15]) performed reliably on PICN and resin nanoceramic blocks but failed to match HF + silane on glass-ceramics. The pilot work by Iliev et al. [51] on hybrid nanoceramic blocks showed that a combination of mild HF etching and silane outperformed universal adhesive alone by 4–7 MPa, even on resin-rich substrates. Albar et al. [52] confirmed the persistent benefit of MDP-based primers when bonding highly translucent zirconia to occlusal dentin. At the same time, Seker and Okutan [42] demonstrated that self-etching primer (Monobond Etch and Prime) approaches HF + air-abrasion on resin-based CAD/CAM blocks but remains inferior on LS_2_. A flowable composite used as an alternative to conventional resin cement on hybrid blocks delivered comparable micro-shear bond strength values, thereby opening a potentially simplified chairside cementation pathway [39]. The effects of resin cement chemistry on the fracture resistance and marginal integrity of chairside CAD/CAM restorations [17,29,30,31,33,37] are summarised in Supplementary Table S6. Demir et al. [56] and Gökay and Aladag [57] additionally documented that SBS values vary considerably between newly introduced ceramic and PEEK-based veneer blocks, supporting material-specific calibration of the cementation protocol.

### 3.7. Clinical Performance and Long-Term Outcomes

Only one prospective clinical study met the eligibility criteria. Adson et al. [26] evaluated chairside CAD/CAM indirect restorations associated with deep margin elevation over a 24-month follow-up period and reported a survival rate of 97.4%, with no debonding events and marginal discoloration in 5.1% of restorations. Because the clinical evidence was limited to a single cohort, no comparisons between material classes could be performed.

Marginal gap measurements remained below 100 µm in most included studies, regardless of material family, when an adequate adhesive protocol was applied [4,17]. Burkhardt et al. [4] reported equivalent retention and marginal integrity for monolithic CAD/CAM crowns adhesively cemented to titanium-base abutments across LS_2_, PICN and zirconia, supporting the use of CAD/CAM blocks in implant-supported single crowns. Mueller et al. [33] showed that immediate dentin sealing (IDS) significantly improved the fatigue resistance of thin occlusal CAD-CAM glass-ceramic and resin-composite veneers, while Fathey et al. [37] confirmed adequate fracture resistance of aesthetic CAD-CAM post-and-core restorations, broadening the indication of chairside CAD/CAM workflows to endodontically compromised teeth.

### 3.8. Biofilm, Biocompatibility, and Influence of Oral Environment

Primary studies examining biological and environmental outcomes indicated that surface characteristics, rather than bulk composition alone, may influence biofilm accumulation and cytocompatibility. Polished monolithic zirconia and lithium disilicate consistently displayed the lowest Streptococcus mutans adhesion in in vitro models, while resin-matrix blocks tended to accumulate thicker biofilms when not properly polished. Mavriqi et al. [13] confirmed the favourable biocompatibility of LS_2_ and ZLS blocks on human gingival fibroblasts. At the same time, da Rosa et al. [21] demonstrated that prophylactic protocols do not significantly compromise the fatigue strength of contemporary CAD/CAM blocks. The work of Piszko et al. [44] on nanometric copper coatings further suggests that tailored surface modifications can simultaneously reduce bacterial adhesion and preserve cytocompatibility, opening new avenues for antibacterial CAD/CAM blocks. Sert et al. [10] additionally documented that surface treatment and thermomechanical ageing modulate the elution of structural ions from CAD/CAM ceramics, with implications for both biocompatibility and long-term mechanical integrity.

To ensure transparent and complete reporting, the review was prepared in accordance with the PRISMA 2020 recommendations. The completed checklist, indicating the location of each required reporting item within the manuscript and supplementary materials, is provided in Supplementary Table S7.

## 4. Discussion

### 4.1. From Material Properties to Clinical Behaviour

The body of evidence retrieved between 2021 and 2026 confirms and refines a set of material-specific principles to guide chairside indirect restorations. Glass-ceramics—and, in particular, lithium disilicate-based blocks—represent the most balanced choice for most aesthetic and posterior indications, combining favourable optical properties, robust flexural strength, and a well-characterised adhesive protocol [1,29]. Advanced lithium disilicate (ALD) blocks, such as Tessera, close the historical mechanical gap between LS_2_ and zirconia, with reported flexural-strength values approaching 500 MPa and fracture resistance equivalent to or superior to that of zirconia after thermocycling, as recently shown by Balladares et al. [29] and Rojas-Rueda et al. [32].

Y-TZP zirconia retains its dominance in stress-bearing sites and extended monolithic restorations, with 36–60-month survival rates consistently above 94% across published clinical cohorts [58]. The recent systematic review and meta-analysis by Alrabiah [8], together with the comparative review by Zeng et al. [59], further demonstrate that CAD/CAM-milled zirconia continues to outperform additively manufactured (3D-printed) zirconia in biaxial flexural strength and fracture toughness, although the gap is narrowing. The recent finite-element and step-stress fatigue work by Nasr et al. [30] highlights that, beyond bulk material strength, preparation design exerts a comparable influence on the long-term fatigue survival of partial-coverage restorations—a finding with direct chairside implications. This concept is also supported by virtual biomechanical simulations of Class II cavity restorations, which emphasise the role of cavity geometry and stress distribution in the mechanical behaviour of restorative materials [60].

### 4.2. Adhesive Strategies—Convergence and Persistent Gaps

The convergence of the included adhesion studies on a material-specific decision tree is one of the most actionable findings of this systematic review. Hydrofluoric acid etching followed by silanisation remains the etch-and-rinse gold standard for glass-ceramics, with baseline shear bond strengths of 26–34 MPa and 20–30 MPa after thermocycling [12,16,42,54]. For zirconia, the synergistic combination of fine-particle alumina air abrasion and an MDP-containing primer is now firmly established as the protocol of choice [16,41,52]. The promise of universal self-etch adhesives to simplify the chairside workflow is partially fulfilled, as follows: they perform reliably on PICN and resin nanoceramic blocks [12,15], but cannot yet replace dedicated HF + silane conditioning on glass-ceramics nor MDP-based protocols on zirconia. The systematic review and meta-analysis of Calheiros-Lobo et al. [54] supports this differential, showing that self-adhesive resin cements are reliable on resin-matrix CAD/CAM blocks but suboptimal on silicate ceramics. Ustun and Ayaz [7] further documented a consistent decrease in bond strength after thermocycling, regardless of cement chemistry, highlighting the need for standardised ageing protocols before bond-strength data can be directly translated into clinical recommendations. The exploratory work of Hassanien et al. [39] on flowable composite as an alternative to resin cement on hybrid blocks merits independent replication. It represents a potentially attractive simplification for chairside cementation, in line with the broader trend toward minimally invasive adhesive restorations, even for endodontically compromised teeth [61]. Likewise, the demonstrations by Chitpattanakul et al. [40] and Albergardi et al. [9] that aged or repaired CAD/CAM blocks can be reliably reconditioned with appropriate solvent-flowable composite combinations support a more conservative repair-first approach to long-term provisional and post-orthodontic management.

### 4.3. Ageing, Surface Stability, and the Role of Polishing

Differences in long-term behaviour between PICN and resin-nanoceramic blocks are now sufficiently consistent across studies to inform clinical recommendations. The data of Fajardo et al. [20] demonstrate that PICN preserves a substantially larger fraction of its initial Vickers microhardness after 10,000 thermal cycles than nano-filled resin composites—directly supporting Hypothesis 1 in our Research Agenda (Section 4.8). For ZLS, the data of Rea et al. [19] establish post-crystallisation polishing as the preferred chairside finishing protocol over conventional glazing, a clinically convenient finding for a single-visit workflow. The simultaneous thermocycling–staining experiment of Elsahn et al. [22] further indicates that hybrid blocks are more susceptible than feldspathic ceramics to ΔE drift after external bleaching, an aspect to be discussed with patients before bleaching of teeth restored with hybrid CAD/CAM blocks is undertaken.

### 4.4. Sources of Heterogeneity Across Included Studies

A recurring feature of the included evidence base is its methodological heterogeneity, which both explains the divergent numerical values reported across studies and justifies the decision not to pool data quantitatively. Several distinct sources of variation can be identified. First, mechanical outcomes were measured with non-equivalent test configurations, as follows: some studies used a biaxial flexural-strength test (piston- or ball-on-three-balls), whereas others used three-point bending, and the two configurations are known to yield systematically different absolute values for the same material, so that flexural-strength figures cannot be compared at face value across studies. Second, adherence to the ISO 6872 standard for dental ceramics—including specimen geometry, surface finish, and span-to-thickness ratio—varied, with several studies applying custom or manufacturer-specific protocols that further limit direct comparability [62]. Third, thermomechanical ageing protocols differed widely, ranging from 5000 to 10,000 thermal cycles and from approximately 50 to 200 N of masticatory load, applied either sequentially or simultaneously, so that “aged” specimens were not subjected to equivalent challenges across studies [31,34,41]. Fourth, specimen preparation, storage medium, and post-bonding conditioning—including immediate dentin sealing, saliva contamination, and try-in cleaning—were inconsistently reported and are themselves known to modulate bond strength by tens of per cent [33,53]. Finally, brand- and generation-specific differences in microstructure and composition within a single nominal material class, together with operator-dependent application of surface treatments, add further variability. Taken together, these considerations imply that between-study differences should be interpreted primarily as a consequence of protocol heterogeneity rather than of genuine material superiority, and they reinforce the need for standardised, ISO-referenced reporting of testing and ageing parameters in future studies.

### 4.5. Positioning Within the Existing Systematic Review Literature

Positioning the present synthesis against the existing secondary literature further strengthens its conclusions. Our finding that fine-particle Al_2_O_3_ air abrasion combined with an MDP-containing primer maximises and stabilises bond strength to zirconia is consistent with the direction of the meta-analytic evidence summarised by Calheiros-Lobo et al. [54], who reported that self-adhesive resin cements remain reliable on resin-matrix CAD/CAM blocks but suboptimal on silicate and zirconia substrates, where MDP-based conditioning is required. Likewise, the persistent superiority of hydrofluoric-acid etching followed by silanisation on glass-ceramics, observed across the primary bonding studies retrieved here, aligns with the established etch-and-rinse paradigm confirmed in earlier syntheses [7,54]. For zirconia mechanical performance, our observation that CAD/CAM-milled zirconia continues to outperform additively manufactured zirconia is in direct agreement with the systematic review and meta-analysis of Alrabiah [8] and the comparative review of Zeng et al. [59]. Where the present work extends beyond these reviews is in their integration, as follows: whereas previous systematic reviews have typically addressed either mechanical behaviour, adhesion, or clinical survival in isolation, this review maps the three layers jointly for the specific context of chairside single-visit restorations. The residual discrepancies between our qualitative synthesis and individual pooled estimates are attributable chiefly to the heterogeneity discussed in Section 4.4—in particular, differences in ageing protocols, bonding substrate (dentin versus enamel versus resin core), and post-bonding storage—rather than to genuine disagreement about the underlying material behaviour.

### 4.6. Clinical Translation

Despite the field’s maturity in mechanics and adhesion, robust long-term clinical evidence remains the limiting factor. The two-year prospective study by Adson et al. [26] on deep-margin elevation combined with chairside CAD/CAM restorations is one of the few clinical studies in the included corpus, and its 97.4% survival rate is encouraging. However, the predominance of in vitro studies—even within highly relevant subdomains such as ALD veneers [32] or repairability of provisional blocks [63]—underscores the need for prospective multicentre cohorts powered to detect material-specific failure modes over five-year horizons. Clinical preference data complement the mechanical evidence, as follows: surveys of practitioner habits regarding adhesive systems for indirect restorations consistently identify ease of use and predictability as the key drivers of material adoption, often ahead of incremental performance differences.

These considerations warrant an explicit statement on the strength of the evidence underlying our recommendations. The great majority of the included studies are in vitro investigations, and only a single prospective clinical cohort met the eligibility criteria. Consequently, the material- and protocol-specific recommendations formulated in this review should be read as evidence-informed considerations for chairside decision making, grounded in mechanistic data and validated surrogate endpoints (flexural strength, bond strength, and fatigue resistance), rather than as definitive clinical-practice guidelines. Wherever a recommendation rests predominantly on laboratory data, this is stated explicitly, and clinical confirmation through adequately powered, prospective multicentre trials with longer follow-up is identified as a prerequisite before the recommendation can be regarded as established.

### 4.7. Gaps in Research

1. The predominance of in vitro studies over prospective clinical investigations is the most prominent limitation of the present evidence base, particularly for the new generation of resin nanoceramic and PICN blocks [6,32,55,64].
2. Ageing and bond-strength testing protocols remain heterogeneous across studies, severely limiting cross-study comparison and preventing formal meta-analysis of mechanical and adhesive outcomes [57].
3. Data on biofilm formation and biocompatibility are largely in vitro, with very few studies replicating the dynamic salivary and dietary environment of the oral cavity [11,13,44].
4. The cumulative impact of prophylactic protocols, dietary acids, and bleaching agents on long-term restoration integrity has been studied predominantly in isolation; combined-stressor protocols are needed [22].
5. Long-term clinical evidence on advanced lithium disilicate (ALD) blocks and on multi-cubic 4Y/5Y zirconias is currently insufficient, despite their rapid market adoption [29,43]. In particular, prospective multicentre clinical trials with follow-up beyond five years are required before the favourable in vitro behaviour of ALD blocks and multilayer (4Y/5Y) zirconias can be translated into evidence-based clinical recommendations.

### 4.8. Research Hypotheses for the Next Five Years

**H1.**  *PICN-based blocks may exhibit a slower decline in mechanical properties after thermomechanical ageing than resin-matrix blocks; whether this translates into improved clinical performance requires prospective investigation [20].*

**H2.**  *Material-specific surface conditioning—hydrofluoric-acid etching followed by silanisation for silica-based glass-ceramics and alumina air abrasion followed by an MDP-containing primer for zirconia—may provide greater long-term bond durability than simplified universal protocols [14,16,18,42,43,50].*

**H3.**  *In stress-bearing posterior sites, zirconia restorations may show different long-term survival and complication profiles compared with hybrid CAD/CAM blocks; direct comparative clinical trials are required [6,8].*

**H4.**  *Post-crystallisation polishing of ZLS ceramics may produce lower surface roughness than pre-crystallisation finishing or conventional glazing; its effect on antagonist wear requires further investigation [19].*

**H5.**  *Universal self-etch adhesives do not match the bond strength of etch-and-rinse/silane systems on glass-ceramics but provide comparable performance on hybrid CAD/CAM blocks [15].*

### 4.9. Strengths and Limitations of This Review

The strengths of this systematic review include reporting in accordance with PRISMA 2020, transparent eligibility and appraisal procedures, and the integrative evaluation of mechanical, adhesive, physico-chemical, and clinical evidence related to chairside CAD/CAM indirect restorations. The interpretation of the present findings should take into account the methodological quality of the included evidence. Although most included studies were rated as having a low or moderate risk of bias, a substantial proportion of the evidence was derived from in vitro investigations, many of which did not report sample size calculations or assessor blinding. Therefore, the favourable mechanical and adhesive behaviour observed for lithium disilicate, advanced lithium disilicate, zirconia, and PICN materials should be interpreted as robust mainly at the laboratory-evidence level, rather than as definitive long-term clinical superiority. Studies rated as high risk of bias were not used to drive the main conclusions but were considered only as supportive or exploratory evidence. This reinforces the need for standardised in vitro protocols and well-designed prospective clinical studies with longer follow-up. The search strategy was structured and reproducible, being conducted in the Web of Science Core Collection and MEDLINE via PubMed and complemented by Google Scholar and reference-list searching. Limitations include the restriction to English-language publications, the absence of prospective protocol registration, and the inability to conduct a quantitative meta-analysis due to substantial heterogeneity across ageing protocols, bond-strength testing methods, material categories, and outcome measures. In addition, Scopus was not searched separately, which may represent a limitation of the search strategy. This limitation was mitigated by using two complementary bibliographic databases and conducting supplementary manual searches to reduce the risk of missing relevant studies. Future updates of this review should incorporate prospective clinical cohorts expected to report between 2026 and 2028 on advanced lithium disilicate and multilayer zirconia blocks.

## 5. Conclusions

Within the limitations of this PRISMA 2020-compliant systematic review of 31 primary studies published between 2021 and 2026, the following conclusions can be drawn:

1. The laboratory evidence indicates favourable flexural-strength and fracture-toughness profiles for lithium disilicate, advanced lithium disilicate, and Y-TZP zirconia. The limited short-term clinical evidence is encouraging but does not permit definitive comparisons of survival between material classes.
2. Under the investigated in vitro thermomechanical ageing conditions, PICN blocks showed greater retention of selected mechanical properties than resin-matrix blocks; however, clinical superiority has not been established.
3. The current adhesion evidence supports hydrofluoric-acid etching followed by silanisation as the most effective conditioning for glass-ceramics, whereas bonding to zirconia is best achieved with alumina air abrasion combined with MDP-containing primers.
4. Universal self-etch adhesives appear to perform acceptably on PICN and resin nanoceramic blocks but, on the basis of the available in vitro data, cannot yet be recommended as a substitute for dedicated surface conditioning on glass-ceramics and zirconia.
5. Standardised ageing protocols, adequately powered prospective multicentre clinical trials, and combined-stressor in vitro models are needed to consolidate the evidence on advanced lithium disilicate and multilayer zirconia blocks before firm long-term clinical recommendations can be made.

Taken together, the present synthesis supports an indication-driven, material-specific chairside protocol—from preparation design to surface treatment, cementation, and finishing—in which the selection of a CAD/CAM block must integrate intrinsic mechanical and physico-chemical properties with the patient-specific loading environment and the operator’s adhesive workflow.

## Figures and Tables

**Table 1:** Bibliographic characteristics of the primary studies included in the qualitative synthesis.

No.	Ref. [n]	Authors	Year	Article Title	Journal	Vol (Issue)	Pages/Art. No.	DOI	Document Type	WoS Accession (UT)
1	[29]	Balladares AO; Chauca-Bajaña L; Balladares MAO; Villafuerte RFD; Carpio-Cevallos C; Jurado CHP; Matías ECO; Armas GV; Suarez-Palacios J; Ron BV	2026	Influence of crystallization and adhesive cementation on the fracture resistance of CAD/CAM advanced lithium disilicate and zirconia-reinforced lithium silicate ceramics after thermocycling	Frontiers in Dental Medicine	7	Art. 1487612	10.3389/fdmed.2026.1715080	Article	WOS:001694019100001
2	[17]	Farag D; El-Damanhoury HM; Abdelaal HM	2026	Marginal Gap and Fracture Strength of Hybrid, Zirconia-Reinforced, and Virgilite-Reinforced CAD/CAM Ceramic Crowns	European Journal of Dentistry	20	134–142	10.1055/s-0046-1820080	Article; Early Access	WOS:001754164600001
3	[30]	Nasr DM; Abdelraheem IM; Watts DC; Silikas N; Borba M; Alharbi N; Althaqafi KA; Elraggal A	2025	Effect of preparation designs and CAD-CAM materials on step-stress fatigue survival of premolar partial coverage restorations: An in-vitro study with fractographic analysis	Dental Materials	41	1018–1029	10.1016/j.dental.2024.11.003	Article	WOS:001400939700001
4	[31]	Rizzatto LV; Meneghetti D; Di Domenico M; Facenda JC; Weber KR; Corazza PH; Borba M	2023	Effect of the type of resin cement on the fracture resistance of chairside CAD-CAM materials after aging	Journal of Advanced Prosthodontics	15	215–224	10.4047/jap.2023.15.3.136	Article	WOS:001035360700004
5	[13]	Mavriqi L; Valente F; Murmura G; Sinjari B; Macrì M; Trubiani O; Caputi S; Traini T	2022	Lithium disilicate and zirconia reinforced lithium silicate glass-ceramics for CAD/CAM dental restorations: biocompatibility, mechanical and microstructural properties after crystallization	Journal of Dentistry	119	Art. 104072	10.1016/j.jdent.2022.104072	Article	WOS:000793705300003
6	[32]	Rojas-Rueda S; Watanabe H; Abuhammoud S; Jurado CA; Alshehri A; Fu CC; Vegh D; Aldosary KM; Algamaiah H; Alshabib A	2025	Fracture resistance of chairside cad/cam advanced lithium disilicate maxillary canine veneers with different incisal edge designs	Saudi Dental Journal	37	245–253	10.1007/s44445-025-00029-8	Article	WOS:001520889200003
7	[23]	Balladares AO; Abad-Coronel C; Ramos JC; Fajardo JI; Paltán CA; Biedma BJM	2024	Comparative Study of the Influence of Heat Treatment on Fracture Resistance of Different Ceramic Materials Used for CAD/CAM Systems	Materials	17	Art. 1246	10.3390/ma17061246	Article	WOS:001192967600001
8	[25]	Fouda AM; Bourauel C; Samran A; Kassem AS; Alhotan A	2024	Effect of glazing and thermocycling on the fracture toughness and hardness of a New fully crystallized aluminosilicate CAD/CAM ceramic material	Bmc Oral Health	24	Art. 619	10.1186/s12903-024-04398-0	Article	WOS:001234542800012
9	[33]	Mueller B; Pilecco RO; Valandro LF; Ruschel VC; Pereira GKR; Bernardon JK	2023	Effect of immediate dentin sealing on load-bearing capacity under accelerated fatigue of thin occlusal veneers made of CAD-CAM glass-ceramic and resin composite material	Dental Materials	39	372–382	10.1016/j.dental.2023.03.003	Article	WOS:000969821000001
10	[34]	Machry RV; Borges ALS; Pereira GKR; Kleverlaan CJ; Venturini AB; Valandro LF	2021	Influence of the foundation substrate on the fatigue behavior of bonded glass, zirconia polycrystals, and polymer infiltrated ceramic simplified CAD-CAM restorations	Journal of the Mechanical Behavior of Biomedical Materials	117	Art. 104387	10.1016/j.jmbbm.2021.104391	Article	WOS:000635269700002
11	[35]	Shono N; Elhejazi A; Maawadh A; Al Nahedh H	2022	Ball-on-three-balls Biaxial Flexural Strength of Bonded and Unbonded CAD/CAM Materials	Ceramics-silikaty	66	66–77	10.13168/cs.2022.0001	Article	WOS:000746650400003
12	[36]	Kawajiri Y; Ikeda H; Nagamatsu Y; Masaki C; Hosokawa R; Shimizu H	2021	PICN Nanocomposite as Dental CAD/CAM Block Comparable to Human Tooth in Terms of Hardness and Flexural Modulus	Materials	14	Art. 1182	10.3390/ma14051182	Article	WOS:000628388000001
13	[37]	Fathey IT; Azer AS; Abdelraheem IM	2024	Fracture resistance and failure mode of three esthetic CAD-CAM post and core restorations	Bmc Oral Health	24	Art. 525	10.1186/s12903-024-04273-y	Article	WOS:001225960700006
14	[15]	Bashary N; Brewster J; Gill P; Janal MN; Oezcan M; Husain NA; Zhang Y	2024	Long-Term Bonding Efficacy of CAD/CAM Hybrid Restorative Materials and Universal Adhesives	European Journal of Prosthodontics and Restorative Dentistry	32(3)	270–276	10.1922/EJPRD_2455Bashary07	Article	WOS:001307084500004
15	[16]	Yavuz SA; Erturk-Avunduk AT; Sagsoz O; Delikan E; Karatas O	2026	Shear Bond Strength of Additively and Subtractively Manufactured CAD/CAM Restorative Materials After Different Surface Treatments and Adhesive Strategies: An In Vitro Study	Polymers	18	Art. 134	10.3390/polym18020296	Article	WOS:001672097800001
16	[18]	González-Angulo E; Fernández-Estevan L; Casas-Terrón J; Senent-Vicente G; Fons-Badal C; Bonmatí FGS; Agustín-Panadero R; Román-Rodríguez JL	2023	Microtensile Bond Strength of CAD-CAM Restorative Dental Material Blocks to Resin Cement: An In Vitro Study	Materials	16(13)	Art. 4796	10.3390/ma16134796	Article	WOS:001028147500001
17	[12]	Vidal CMP; Teixeira EC; Armstrong SR; Qian F	2024	Comparison of Adhesion Performance of a Self-curing and a Light-curing Universal Adhesive to Various Dental Substrates and CAD/CAM Materials	Journal of Adhesive Dentistry	26	31–40	10.3290/j.jad.b4908469	Article	WOS:001157950100002
18	[38]	Alper SB; Tekçe N; Fidan S; Balci S	2023	Comparison of the wear behavior and surface properties of zirconia and resin-based CAD/CAM restorative materials after different sandblasting procedures	Surface Topography-metrology and Properties	11	Art. 035017	10.1088/2051-672X/acb8cd	Article	WOS:000929461400001
19	[39]	Hassanien EEY; Tolba ZO	2024	Flowable composite as an alternative to adhesive resin cement in bonding hybrid CAD/CAM materials: in-vitro study of micro-shear bond strength	Bdj Open	10	Art. 41	10.1038/s41405-024-00251-2	Article	WOS:001294464100001
20	[40]	Chitpattanakul P; Prawatvatchara W; Limpuangthip N; Katheng A; Uasuwan P; Boonpitak K	2025	Effect of various solvents on the repairability of aged CAD/CAM provisional restorative materials with flowable resin composite: an in vitro study	Bmc Oral Health	25	Art. 198	10.1186/s12903-025-05731-x	Article	WOS:001442745600001
21	[41]	Alper SB; Tekçe N; Yildirim ES; Sancak EI; Fidan S	2023	The effect of different parameters on the wear, surface properties, and shear bond strength of monolithic zirconia CAD/CAM restorative materials	Journal of Adhesion Science and Technology	37	2891–2908	10.1080/01694243.2023.2194069	Article	WOS:000960317800001
22	[42]	Seker H; Okutan Y	2024	Self-etching primer versus hydrofluoric acid and air-abrasion: effects on flexural and resin bond strengths of resin-based CAD/CAM material	Journal of Adhesion Science and Technology	38	2731–2748	10.1080/01694243.2024.2313373	Article	WOS:001162131400001
23	[43]	Pulido C; Prieto-Torres O; Carvajal-Garzón C; Dávila-Sánchez A; Gonzalez-Vaca C; Arrais CAG	2026	Prolonged application methods of self-etching primer: Effect of aging on micro-shear bond strength to lithium disilicate and zirconia-reinforced lithium silicate ceramics	Journal of Prosthodontics-implant Esthetic and Reconstructive Dentistry	35	585–594	10.1111/jopr.70085	Article	WOS:001659233400001
24	[26]	Adson O; Sarikaya TY; Bolay S	2026	Clinical evaluation of deep margin elevation in CAD/CAM indirect restorations: A 24-month follow-up study	Journal of Dentistry	145	Art. 104952	10.1016/j.jdent.2026.106522	Article	WOS:001691662600001
25	[19]	Rea FT; Valcanaia A; Herrera-Fierro P; Verma M; Neiva GD	2024	Surface Roughness Evaluation of Pre- Versus Post-Crystallization Polish of Two High-Strength Silicate Ceramics for Chairside CAD/CAM Technology	Applied Sciences-basel	14	Art. 2768	10.3390/app14072768	Article	WOS:001201529600001
26	[20]	Fajardo JI; Paltán CA; Leon M; Matute AY; Armas-Vega A; Puratambi RH; Delgado-Gaete BA; Requena S; Benalcazar A	2025	Impact of Thermal Cycling on the Vickers Microhardness of Dental CAD/CAM Materials: Greater Retention in Polymer-Infiltrated Ceramic Networks (PICNs) Compared to Nano-Filled Resin Composites	Ceramics-switzerland	8	Art. 35	10.3390/ceramics8020035	Article	WOS:001646235600001
27	[21]	da Rosa LS; Souza LFB; Pilecco RO; Kluch TAC; Binotto FS; Henriques VZ; Kleverlaan CJ; Pereira GKR; Tribst JPM	2024	Are Dental Prophylaxis Protocols Safe for CAD-CAM Restorative Materials? Surface Characteristics and Fatigue Strength	Coatings	14	Art. 715	10.3390/coatings14060715	Article	WOS:001385447700001
28	[22]	Elsahn NA; Allawi NM; Maged A; Alkhatab AL; Cahyanto A	2026	Effect of 32% Hydrogen Peroxide Bleaching on Surface Properties and Staining Susceptibility of CAD/CAM Hybrid and Feldspathic Ceramics Under Simultaneous Thermocycling-Staining Challenge	European Journal of Dentistry	20	211–220	10.1055/s-0046-1816535	Article; Early Access	WOS:001690010900001
29	[24]	Sahin O; Köroglu A; Dede DÖ; Yildirim H; Yagci Ü; Erdal SG	2025	Effect of Different Surface Treatments and Toothbrushing Durations on Surface Roughness and Color Stability of CAD/CAM Interim Crown Material	Coatings	15	Art. 1377	10.3390/coatings15121377	Article	WOS:001648200000001
30	[44]	Piszko A; Grzebieluch W; Piszko PJ; Rusak A; Pajaczkowska M; Nowicka J; Kobielarz M; Mikulewicz M; Dobrzynski M	2024	Cytotoxicity and Microbiological Properties of Ceramic CAD/CAM Materials Subjected to Surface Treatment with Nanometric Copper Layer	Applied Sciences-basel	14	Art. 9224	10.3390/app14209224	Article	WOS:001341678300001
31	[10]	Sert M; Çakmak G; Subasi MG; Donmez MB; Yilmaz B	2022	Effect of different surface treatments and thermomechanical aging on the ion elution of CAD-CAM materials	Journal of Prosthetic Dentistry	127(6)	926.e1–926.e10	10.1016/j.prosdent.2022.03.021	Article	WOS:000840261200017

**Table 2:** Composition, microstructure, and main clinical indications of representative chairside CAD/CAM blocks (*n* = 11 representative products).

Family	Representative Product (Manufacturer)	Microstructure/Composition	Main Clinical Indication
Feldspathic	Vita Mark II (Vita Zahnfabrik, Bad Säckingen, Germany)	Fine-particle feldspathic glass (~4 µm)	Veneers, anterior crowns, low-stress inlays
Leucite-reinforced	IPS Empress CAD (Ivoclar Vivadent AG, Schaan, Liechtenstein)	35–45 vol% leucite crystals in glass matrix	Anterior crowns, inlays, and onlays
Lithium disilicate (LS_2_)	IPS e.max CAD (Ivoclar Vivadent AG, Schaan, Liechtenstein)	70 vol% Li_2_Si_2_O_5_ crystals in glass matrix	Anterior and posterior crowns, onlays, 3-unit FPDs
Advanced lithium disilicate (ALD)	Tessera/CEREC Tessera (Dentsply Sirona, Charlotte, NC, USA)	Virgilite-reinforced LS_2_ glass-ceramic	Posterior crowns, monolithic onlays
Zirconia-reinforced lithium silicate (ZLS)	VITA Suprinity (Vita Vivadent AG, Schaan, Liechtenstein)/Celtra Duo (DeguDent GmbH, Hanau-Wolfgang, Germany)	10 wt% ZrO_2_ dissolved in LS glass-ceramic	Anterior and posterior crowns, veneers
3Y-TZP zirconia	Katana HT (Kuraray, Noritake Dental Inc., Tokyo, Japan)	Y-TZP (~3 mol% Y_2_O_3_) tetragonal polycrystals	Posterior monolithic crowns, FPDs
4Y/5Y-TZP zirconia	Katana STML/UTML (Kuraray Noritake Dental Inc., Tokyo, Japan), Lava Aesthetic (3M Oral Care, St. Paul, MN, USA)	Multi-cubic Y-TZP (4–5 mol% Y_2_O_3_)	Anterior and posterior monolithic crowns
PICN	Vita Enamic (VITA Zahnfabrik H. Rauter GmbH & Co. KG, Bad Säckingen, Germany)	86 wt% feldspathic ceramic + 14 wt% UDMA/TEGDMA	Inlays, onlays, endo-treated teeth, and implant crowns
Resin nanoceramic	Lava Ultimate (3M ESPE, St. Paul, MN, USA)	80 wt% silica/zirconia nanoclusters in UDMA	Inlays, onlays, single crowns (off-label)
Resin nanoceramic	GC Cerasmart 270 (GC Dental Products Corp., Kasugai, Aichi, Japan)	71 wt% Ba-glass + silica nanoparticles in resin	Inlays, onlays, partial crowns, implant crowns
Resin composite (CAD/CAM)	Brilliant Crios (Coltene/Whaledent AG, Altstätten, Switzerland)	70.7 wt% Ba-glass + amorphous silica in cross-linked resin	Provisional and long-term restorations, repairs

**Table 3:** Descriptive extracted ranges of mechanical and physico-chemical properties of chairside CAD/CAM materials, as reported in the included studies. Values represent minimum–maximum ranges identified across eligible studies and were not statistically pooled.

Material	σ_flex (MPa)	K_IC (MPa·m^0.5^)	E (GPa)	HV (GPa)	Water Sorption (μg/mm^3^)
Feldspathic	100–130	1.0–1.3	40–50	5.5–6.5	20–25
Leucite-reinforced	150–180	1.2–1.5	60–70	5.0–5.8	23–28
PICN	150–170	1.4–1.7	28–35	2.5–3.0	25–32
Resin nanoceramic	180–230	1.7–2.1	12–18	1.0–1.4	32–42
ZLS (Suprinity/Celtra)	210–280	2.0–2.5	65–75	6.6–7.2	16–22
Lithium disilicate (e.max CAD)	330–410	2.5–3.1	90–100	5.8–6.4	12–16
ALD (Tessera)	445–520	2.8–3.2	95–105	6.5–7.0	11–15
3Y-TZP	1000–1200	5.0–6.0	200–220	12–13	<5
5Y-TZP	650–820	3.5–4.5	190–210	11–12	<5

**Table 4:** Effect of thermomechanical ageing on representative chairside CAD/CAM materials. TC = thermal cycles; MC = mechanical cycles.

Material	Ageing Protocol	ΔHV (%)	Δσ_flex (%)	Δ Surface Roughness Ra
Feldspathic	5000 TC + 1.2 M MC	−7 to −10	−8 to −12	+0.05–0.10 µm
Lithium disilicate	10,000 TC + 1.2 M MC	−3 to −6	−4 to −8	+0.02–0.06 µm
ALD	10,000 TC + 1.2 M MC	−2 to −5	−3 to −6	+0.02–0.05 µm
ZLS	5000 TC + 1.2 M MC	−4 to −7	−5 to −9	+0.04–0.08 µm
3Y-TZP	10,000 TC + 2 M MC	−1 to −3	−2 to −5	+0.01–0.03 µm
PICN	5000 TC + 1.2 M MC	−9 to −13	−7 to −11	+0.10–0.18 µm
Resin nanoceramic	5000 TC + 1.2 M MC	−22 to −30	−15 to −22	+0.20–0.35 µm

Note: numerical values are unweighted descriptive ranges (minimum–maximum) or representative mid-range values extracted from the included primary studies; because of substantial heterogeneity in study design, specimen geometry, ageing protocols and testing methods, no meta-analytic pooling was performed, and these values should be interpreted only as qualitative benchmarks for comparison between material families, not as pooled effect estimates.

**Table 5:** Surface-conditioning and adhesive cementation protocols most consistently supported by the included laboratory evidence.

Material	Surface Treatment Most Consistently Supported by Laboratory Evidence	Coupling Agent/Primer	Cement Chemistry
Feldspathic/leucite	5–9% HF, 60–90 s	Silane primer	Light- or dual-cure resin cement
Lithium disilicate/ZLS/ALD	5% HF, 20 s + ultrasonic cleaning	Silane primer (or MDP-silane combo)	Dual-cure resin cement
3Y-TZP zirconia	Al_2_O_3_ 50 µm, 2–3 bar	MDP-containing primer	MDP-containing resin cement
4Y/5Y-TZP zirconia	Al_2_O_3_ 30–50 µm, 2 bar (low-pressure)	MDP-containing primer	Self-adhesive or dual-cure resin cement
PICN	5% HF 60 s (or Al_2_O_3_ 30 µm)	Silane primer	Dual-cure resin cement
Resin nanoceramic	Al_2_O_3_ 30–50 µm + silane (or universal adhesive)	Universal adhesive/silane	Dual-cure resin cement
Resin composite (Brilliant Crios)	Al_2_O_3_ 30 µm + silane	Universal adhesive	Dual-cure resin cement

## Data Availability

No new data were created or analyzed in this study. Data sharing is not applicable to this article.

## Supplementary Materials

The following supporting information can be downloaded at: https://www.mdpi.com/article/10.3390/jfb17080381/s1, Supplementary Tables S1–S7.
