# Supplementary material for: Advances in Chairside and Restorative Dental Materials: A Systematic Review of Material Properties, Adhesive Strategies, and Long-Term Performance
Source: J Funct Biomater. 2026 Aug 3;17(8):381. doi: 10.3390/jfb17080381 (PMC13514366; doi:10.3390/jfb17080381)
Supplement: Supplementary file 1 [file jfb-17-00381-s001.zip › Supplementary Table S7.pdf]

Supplementary Table S7. PRISMA 2020 Checklist

Section and Topic	Item #		Location where item is reported
**TITLE**			
Title	1	Identify the report as a systematic review.	Title, p. 1.
**ABSTRACT**			
Abstract	2	See the PRISMA 2020 for Abstracts checklist.	Structured Abstract, pp. 2–3.
**INTRODUCTION**			
Rationale	3	Describe the rationale for the review in the context of existing knowledge.	Introduction, pp. 3–4.
Objectives	4	Provide an explicit statement of the objective(s) or question(s) the review addresses.	Introduction, final paragraph, p. 4; Section 2.2, p. 5.
**METHODS**			
Eligibility criteria	5	Specify the inclusion and exclusion criteria for the review and how studies were grouped for the syntheses.	Sections 2.2 and 2.4, pp. 5 and 7; studies were grouped by outcome domain in Section 3.1, pp. 9–10.
Information sources	6	Specify all databases, registers, websites, organisations, reference lists and other sources searched or consulted to identify studies. Specify the date when each source was last searched or consulted.	Section 2.3, pp. 5–6 (Web of Science Core Collection, MEDLINE via PubMed, Google Scholar, and reference-list screening; search dates reported).
Search strategy	7	Present the full search strategies for all databases, registers and websites, including any filters and limits used.	Section 2.3, pp. 5–6. The complete Web of Science Core Collection search strategy is reported. The MEDLINE via PubMed search was adapted to the database-specific syntax using the same predefined concepts, eligibility period, and language restriction. The supplementary Google Scholar search approach

Section and Topic	Item #		Location where item is reported
			and stopping rule are also described.
Selection process	8	Specify the methods used to decide whether a study met the inclusion criteria of the review, including how many reviewers screened each record and each report retrieved, whether they worked independently, and if applicable, details of automation tools used in the process.	Section 2.5, p. 7 (two independent reviewers; disagreements resolved by discussion or a third reviewer).
Data collection process	9	Specify the methods used to collect data from reports, including how many reviewers collected data from each report, whether they worked independently, any processes for obtaining or confirming data from study investigators, and if applicable, details of automation tools used in the process.	Section 2.5, p. 7 (standardised extraction form and extracted fields). Two reviewers independently extracted the data using a standardized form.
Data items	10a	List and define all outcomes for which data were sought. Specify whether all results that were compatible with each outcome domain in each study were sought (e.g. for all measures, time points, analyses), and if not, the methods used to decide which results to collect.	Sections 2.2 and 2.5, pp. 5 and 7. Outcome domains and principal numerical findings are reported; the rule used to select among multiple compatible measures or time points is not explicitly stated.
	10b	List and define all other variables for which data were sought (e.g. participant and intervention characteristics, funding sources). Describe any assumptions made about any missing or unclear information.	Section 2.5, p. 7 (study design, sample/specimen characteristics, material family and product, comparator, protocols, ageing procedure, test method, standards, and other study variables).
Study risk of bias assessment	11	Specify the methods used to assess risk of bias in the included studies, including details of the tool(s) used, how many reviewers assessed each study and whether they worked independently, and if applicable, details of automation tools used in the process.	Section 2.7, pp. 9–10; Supplementary Tables S1–S3 (Supplementary Table S1: study-level summary; Supplementary Table S2: appraisal instruments, scoring procedures, and

Section and Topic	Item #		Location where item is reported
			thresholds; Supplementary Table S3: item-level judgements and overall classifications). Two reviewers performed the assessments independently, and inter-rater agreement was evaluated using Cohen's kappa.
Effect measures	12	Specify for each outcome the effect measure(s) (e.g. risk ratio, mean difference) used in the synthesis or presentation of results.	Section 2.6, pp. 8–9. No common comparative effect measure was selected. Results were presented as study-specific numerical values and unweighted descriptive minimum–maximum ranges.
Synthesis methods	13a	Describe the processes used to decide which studies were eligible for each synthesis (e.g. tabulating the study intervention characteristics and comparing against the planned groups for each synthesis (item #5)).	Sections 2.2, 2.4, 2.5, and 2.5.1, pp. 5–8; studies were classified by design, material family, and principal outcome domain.
	13b	Describe any methods required to prepare the data for presentation or synthesis, such as handling of missing summary statistics, or data conversions.	Section 2.6, pp. 8–9 (handling of heterogeneous numerical data, ranges, and graphical mid-range values; no statistical conversions or pooling).
	13c	Describe any methods used to tabulate or visually display results of individual studies and syntheses.	Section 2.6, pp. 8–9; Figures 1–3; Tables 1–5; Supplementary Tables S1–S6.
	13d	Describe any methods used to synthesize results and provide a rationale for the choice(s). If meta-analysis was performed, describe	Sections 2.1 and 2.6, pp. 5 and 8–9. Qualitative

Section and Topic	Item #		Location where item is reported
		the model(s), method(s) to identify the presence and extent of statistical heterogeneity, and software package(s) used.	descriptive synthesis was selected because substantial methodological and outcome heterogeneity precluded meta-analysis.
	13e	Describe any methods used to explore possible causes of heterogeneity among study results (e.g. subgroup analysis, meta-regression).	No formal subgroup analysis or meta-regression was conducted. Sources of heterogeneity were explored narratively in Section 4.4.
	13f	Describe any sensitivity analyses conducted to assess robustness of the synthesized results.	Not applicable. No formal sensitivity analyses were conducted because no meta-analysis or statistical synthesis was performed.
Reporting bias assessment	14	Describe any methods used to assess risk of bias due to missing results in a synthesis (arising from reporting biases).	Not assessed. Risk of bias due to missing results or publication bias was not formally assessed because the synthesis was qualitative and the evidence domains were highly heterogeneous.
Certainty assessment	15	Describe any methods used to assess certainty (or confidence) in the body of evidence for an outcome.	No formal certainty-of-evidence framework (e.g., GRADE) was applied. The strength and limitations of the evidence are discussed narratively in Sections 4.6, 4.7, and 4.9.
**RESULTS**			
Study selection	16a	Describe the results of the search and selection process, from the number of records identified in the search to the number of studies	Section 3.1, p. 9; Figure 1, p. 8.

Section and Topic	Item #		Location where item is reported
		included in the review, ideally using a flow diagram.	
	16b	Cite studies that might appear to meet the inclusion criteria, but which were excluded, and explain why they were excluded.	Figure 1, p. 8, reports the predefined categories and corresponding numbers of full-text exclusions. Individual citation-level details for excluded full-text reports were not retained in a separate supplementary list.
Study characteristics	17	Cite each included study and present its characteristics.	Table 1, pp. 7–8, presents the bibliographic characteristics of all included primary studies; additional study characteristics and outcome-domain grouping are reported in Sections 2.5 and 3.1, pp. 7–10.
Risk of bias in studies	18	Present assessments of risk of bias for each included study.	Section 3.2, p. 10; Supplementary Table S1 provides the study-level summary, and Supplementary Table S3 presents the item-level judgements, total scores, and overall risk-of-bias classifications for each included study.
Results of individual studies	19	For all outcomes, present, for each study: (a) summary statistics for each group (where appropriate) and (b) an effect estimate and its precision (e.g. confidence/credible interval), ideally using structured tables or plots.	Sections 3.4–3.8; Tables 2–5; Supplementary Tables S4–S6. Study-specific findings are reported where available, while descriptive ranges are used when

Section and Topic	Item #		Location where item is reported
			methodological and reporting heterogeneity precluded a common effect estimate. Measures of precision were not consistently reported across the included studies.
Results of syntheses	20a	For each synthesis, briefly summarise the characteristics and risk of bias among contributing studies.	Sections 3.1–3.8. The characteristics of the contributing studies are summarised in Table 1 and by outcome domain in Section 3.1; the corresponding risk-of-bias context is presented in Section 3.2 and Supplementary Tables S1 and S3.
	20b	Present results of all statistical syntheses conducted. If meta-analysis was done, present for each the summary estimate and its precision (e.g. confidence/credible interval) and measures of statistical heterogeneity. If comparing groups, describe the direction of the effect.	Not applicable to meta-analysis. Qualitative descriptive results and directions of findings are presented in Sections 3.3–3.8 and Tables 1–4.
	20c	Present results of all investigations of possible causes of heterogeneity among study results.	Section 4.4 (narrative examination of methodological and clinical heterogeneity).
	20d	Present results of all sensitivity analyses conducted to assess the robustness of the synthesized results.	Not applicable. No sensitivity analyses were conducted.
Reporting biases	21	Present assessments of risk of bias due to missing results (arising from reporting biases) for each synthesis assessed.	Not applicable. Reporting-bias assessments were not conducted.
Certainty of evidence	22	Present assessments of certainty (or confidence) in the body of evidence for each outcome assessed.	No formal certainty assessment was conducted. Narrative

Section and Topic	Item #		Location where item is reported
			qualification of the evidence is provided in Sections 4.6, 4.7, and 4.9.
**DISCUSSION**			
Discussion	23a	Provide a general interpretation of the results in the context of other evidence.	Sections 4.1–4.6, including comparison with previous systematic reviews in Section 4.5.
	23b	Discuss any limitations of the evidence included in the review.	Sections 4.6, 4.7, and 4.9 (limitations of the predominantly in vitro evidence base and limited clinical evidence).
	23c	Discuss any limitations of the review processes used.	Section 4.9 (limitations of the review process, including database coverage, language/date restrictions, and heterogeneity).
	23d	Discuss implications of the results for practice, policy, and future research.	Sections 4.6–4.8 and Section 5 (clinical translation, research gaps, future hypotheses, and conclusions).
**OTHER INFORMATION**			
Registration and protocol	24a	Provide registration information for the review, including register name and registration number, or state that the review was not registered.	Section 2.1, p. 5. Retrospective registration in the Open Science Framework on 13 July 2026; DOI: 10.17605/OSF.IO/26FCD.
	24b	Indicate where the review protocol can be accessed, or state that a protocol was not prepared.	Section 2.1, p. 5. The retrospectively registered protocol is available through the Open Science

Section and Topic	Item #		Location where item is reported
			Framework record (DOI: 10.17605/OSF.IO/26FCD).
	24c	Describe and explain any amendments to information provided at registration or in the protocol.	Not applicable. The protocol was registered retrospectively after completion of the review; therefore, amendments following prospective registration could not be assessed.
Support	25	Describe sources of financial or non-financial support for the review, and the role of the funders or sponsors in the review.	Funding statement, end matter: no external funding. Acknowledgements disclose institutional support for publication costs.
Competing interests	26	Declare any competing interests of review authors.	Conflicts of Interest statement, end matter: the authors declare no conflicts of interest.
Availability of data, code and other materials	27	Report which of the following are publicly available and where they can be found: template data collection forms; data extracted from included studies; data used for all analyses; analytic code; any other materials used in the review.	Data Availability Statement, end matter; extracted study information and appraisal data are reported in Supplementary Tables S1–S5. No analytic code was generated because no meta-analysis was performed.

From: Page MJ, McKenzie JE, Bossuyt PM, Boutron I, Hoffmann TC, Mulrow CD, et al. The PRISMA 2020 statement: an updated guideline for reporting systematic reviews. BMJ 2021;372:n71. doi: 10.1136/bmj.n71. This work is licensed under CC BY 4.0. To view a copy of this license, visit https://creativecommons.org/licenses/by/4.0/
