# Supplementary material for: Advances in Chairside and Restorative Dental Materials: A Systematic Review of Material Properties, Adhesive Strategies, and Long-Term Performance
Source: J Funct Biomater. 2026 Aug 3;17(8):381. doi: 10.3390/jfb17080381 (PMC13514366; doi:10.3390/jfb17080381)
Supplement: Supplementary file 1 [file jfb-17-00381-s001.zip › Supplementary Tables S4-S6.pdf]

Table S4. Optical properties of chairside CAD/CAM materials: translucency parameter (TP), contrast ratio (CR) and colour stability after thermocycling and bleaching, as reported in the included studies [19,22,24,25]. Note: numerical values are unweighted descriptive ranges (minimum–maximum) or representative mid-range values extracted from the included primary studies; because of substantial heterogeneity in study design, specimen geometry, ageing protocols and testing methods, no meta-analytic pooling was performed, and these values should be interpreted only as qualitative benchmarks for comparison between material families, not as pooled effect estimates.

Material	TP (1 mm)	CR	ΔE after 5,000 TC	ΔE after 32% H_2O_2
Feldspathic	20–24	0.55–0.62	0.6–1.0	1.5–2.4
Leucite	18–22	0.60–0.66	0.8–1.2	1.7–2.6
Lithium disilicate	16–20	0.65–0.72	0.5–0.9	1.0–1.8
ALD	14–18	0.70–0.76	0.6–1.0	1.1–1.9
ZLS	15–19	0.68–0.74	0.6–1.1	1.2–2.0
PICN	12–16	0.74–0.80	1.2–1.8	2.6–3.4
Resin nanoceramic	11–15	0.78–0.85	1.6–2.4	3.2–4.5

Note: numerical values are unweighted descriptive ranges (minimum–maximum) or representative mid-range values extracted from the included primary studies; because of substantial heterogeneity in study design, specimen geometry, ageing protocols and testing methods, no meta-analytic pooling was performed, and these values should be interpreted only as qualitative benchmarks for comparison between material families, not as pooled effect estimates.

Table S5. Shear bond strength (SBS) and micro-tensile bond strength (μTBS) values reported for chairside CAD/CAM materials before and after ageing. Compiled from the included studies [12,15,16,18,38,39,41–43].

Material	Surface treatment	SBS / μTBS — baseline (MPa)	After 5,000 TC (MPa)	Failure pattern
Feldspathic	HF + silane	26 ± 4	20 ± 5	Mainly cohesive
Lithium disilicate	HF + silane	32 ± 5	27 ± 4	Mainly cohesive
ALD	HF + silane (MDP-doped)	34 ± 5	30 ± 4	Mixed
3Y-TZP	Al_2O_3 + MDP primer	28 ± 3	24 ± 4	Adhesive/mixed
3Y-TZP	Universal adhesive only	18 ± 4	12 ± 5	Mainly adhesive
PICN	HF + silane	30 ± 4	26 ± 4	Cohesive in resin matrix
Resin nanoceramic	Al_2O_3 + universal adhesive	29 ± 5	23 ± 5	Mixed

Note: numerical values are unweighted descriptive ranges (minimum–maximum) or representative mid-range values extracted from the included primary studies; because of substantial heterogeneity in study design, specimen geometry, ageing protocols and testing methods, no meta-analytic pooling was performed, and these values should be interpreted only as qualitative benchmarks for comparison between material families, not as pooled effect estimates.

Table S6. Effect of resin cement chemistry on the fracture resistance and marginal integrity of chairside CAD/CAM crowns and partial restorations [17,29–31,33,37].

**Cement type**	**Tested material**	**Fracture load (N)**	**Marginal gap (μm)**
Conventional dual-cure resin cement	LS_2 / ALD	1450–1820	60–90
MDP-containing self-adhesive cement	Y-TZP	1900–2400	70–110
Light-cure resin cement	Feldspathic / leucite	650–960	55–85
Self-curing universal adhesive + flowable	PICN / resin nanoceramic	1100–1450	70–100
Glass ionomer (control)	Y-TZP	1450–1750	100–150

Note: numerical values are unweighted descriptive ranges (minimum–maximum) or representative mid-range values extracted from the included primary studies; because of substantial heterogeneity in study design, specimen geometry, ageing protocols and testing methods, no meta-analytic pooling was performed, and these values should be interpreted only as qualitative benchmarks for comparison between material families, not as pooled effect estimates.
